# Rapid Detection of Chloramphenicol Residues in Aquatic Products Using Colloidal Gold Immunochromatographic Assay

**DOI:** 10.3390/s141121872

**Published:** 2014-11-18

**Authors:** Chennan Zhou, Xueyin Zhang, Xinxin Huang, Xishan Guo, Qiang Cai, Songming Zhu

**Affiliations:** 1 College of Biosystems Engineering and Food Science, Zhejiang University, Hangzhou 310058, China; E-Mails: wszcn@126.com (C.Z.); 21213025@zju.edu.cn (X.Z.); zhusm@zju.edu.cn (S.Z.); 2 Shanghai Entry-Exit Inspection and Quarantine Bureau, Shanghai 200000, China; E-Mail: huangxinxin@shciq.gov.cn; 3 Yangtze Delta Region Institute of Tsinghua University, Jiaxing 310013, China; E-Mail: caiq@tsinghua.edu.cn

**Keywords:** chloramphenicol, colloidal gold nanoparticles, fishery drug residues, immunochromatographic assay

## Abstract

A colloidal gold immunochromatographic assay (GICA) was developed for rapid detection of chloramphenicol (CAP) residues in aquatic products. A nitrocellulose (NC) membrane was used as the carrier, and the polyclonal CAP antibody was used as the marker protein. The average diameter of as-prepared colloidal gold nanoparticles (AuNPs) was about 20 nm. The optimal pH value of colloidal gold solutions and the amount of the antibody of CAP were 8.0 and 7.2 μg/mL, respectively. The CAP antibody was immobilized onto the conjugate pad after purification. The CAP conjugate and goat anti-rabbit IgG (secondary antibody) were coated onto the NC membrane. Next, the non-specific sites were blocked with 1% bovine serum albumin. The minimum detectable concentration of CAP in standard solution is 0.5 ng/mL, with good reproducibility. For the real samples from crucian carps injected with a single-dose of CAP in the dorsal muscles, the minimum detectable concentration of CAP residues was 0.5 μg/kg. The chromatographic analysis time was less than 10 min, and the strip had a long storage lifetime of more than 90 days at different temperatures. The strips provide a means for rapid detection of CAP residues in aquatic products.

## 1. Introduction

Worldwide, aquaculture production has grown at an average annual rate of 8.4% since 1970 and reached 65.8 million tons in 2008, of which the aquaculture production of China accounted for 61.5% [1]. However, due to the implementation of high-density rearing, as well as over-feeding, industrial wastewater pollution and natural disasters, aquatic diseases are quite prevalent in China. Thus, antibiotics and chemicals are widely used to ensure high quality aquaculture products. Because of this, the problem of fishery drug residues in aquatic products is of great concern to the Chinese people.

One class of drugs in particular, the chloramphenicol (CAP) drugs, is used extensively in aquaculture due to its broad spectrum activity, ready availability and low cost [2]. CAP is a wide-spectrum antibiotic effective against many Gram-positive and Gram-negative bacteria, including several anaerobic organisms [3]. However, due to a number of dangerous side-effects, chloramphenicol is no longer given to humans unless the bacterial infection is resistant to other antibiotics. Newborn infants receiving high or unmodified doses of chloramphenicol can develop “gray baby syndrome” [4], and potentially fatal side-effects such as aplastic anemia and hypersensitivity are known to occur in human beings [5]. In consideration of the harm that CAP does to humans, many countries, including the USA, Canada, Japan and the EU member states, have banned its use in food animals [6,7]. In 2002, the EU imposed a 30-month ban on shrimp imports from China because of illegal chloramphenicol antibiotic use, and in 2006 the United States rejected shrimp imports from China because of repeated chloramphenicol contamination [8], both of which caused a huge economic impact on China's aquatic production industry. The Chinese government also has banned the use of CAP in aquatic species, but some farmers still use CAP to control fish diseases [9]. Even now, CAP residues are detected frequently in aquatic products by the government supervision departments. Hence, in order to strictly monitor the use of CAP in aquatic products, the fast, simple, sensitive and cost-effective technologies and methods for analysis of CAP residues is essential.

However, currently the standard reference methods for the determination of drug residues in real samples are mainly based on gas chromatography [10,11], mass spectrometry [12], liquid chromatography [13–15] or enzyme-linked immunoassay (ELISA) [16]. Although these methods are sensitive and accurate for detection of CAP residues in fish, they have some disadvantages, such as the need for complicated pretreatments, and time-consuming and costly procedures.

It should be noted that the microbial assay has traditionally been used for detecting antibiotic residues in food products [17,18], and is both simple to perform and relatively less expensive, but it is not widely used in practical applications because of the low sensitivity and time-consuming procedures involved. Nowadays, immune biosensors have increasingly attracted interest in the field of rapid drug residue detection in foods, due to their specificity, simple operation, fast response and low cost [19–21]. This is especially true of the immuno-strip biosensor method, which holds potential for practical applications, since the immune-chromatographic assay is cheap to manufacture; requires a short analysis time (time-to-result no exceeding 20 min); is easy-to-use (just add a drop of sample in the right container); has high sensitivity; and the results can be seen with the naked eye. Immunochromatographic assays based on colloidal gold nanoparticles have been used in hospitals and medical centers for many years [22]. In recent years, colloidal gold immunochromatographic strips have been developed and applied in many regions of food safety, such as for the detection of methamidophos residues [23] and clenbuterol [24]. Besides, an immunochromatographic assay has been developed to determine CAP in milk with a detection limit of 10 ng/mL [25], but this value is well above the maximum residual permisable limit of 0.3 ng/g in animal foods set by the EU and some other countries [26], as well as China. Although a quantum dot-based lateral flow immunoassay was developed to obtain a high sensitivity with a LOD of 0.3 ng/mL, results of the assay could be seen by eye under UV light excitation [27]. Hence, it is still necessary to study the high sensitive, easy-to-use, and low-cost immunochromatographic assay for the determination of CAP residues in aquatic products.

In this study, competitive colloidal gold immunochromatographic strips with high sensitivity, fast response and low cost for on-site detection of CAP residues were developed, and the parameters of the reaction steps and conditions were optimized through modification of the preparation procedures.

## 2. Materials and Methods

### 2.1. Materials

Polyclonal chloramphenicol (CAP) antibody, chloramphenicol and goat anti-rabbit IgG were obtained from Bioss Tech Co (Beijing, China); Chloroauric acid (HAuCl_4_·3H_2_O) was purchased from Jieding Tech Co. (Shanghai, China). Bovine serum albumin (BSA) was obtained from Sigma (Shanghai, China). Analytically pure PEG20000, sucrose, and Tween-20 were applied in the experiments. Nitrocellulose membranes (Millipore 135), fibrous membranes, absorbent pads and PVC backing cards were obtained from Kinbiao Tech Co., Ltd (Shanghai, China). The other chemicals used were of analytical grade.

### 2.2. Apparatus

A UV Probe-2450 spectrometer (Shimadzu Corporation, Tokyo, Japan) was used to characterize the colloidal AuNPs and AuNPs-antibody conjugate. All distilled water used was purified using an ultra-pure water system from Millipore Co. (Bedford, MA, USA). A magnetic stirrer was obtained from Huanyu Scientific Factory (Jiangsu, China). A high-speed freezing centrifuge (H3018DR) was bought from Zhixin Experimental Instrument Technology Co. (Shanghai, China). A transmission electron microscope (TEM) (JEOL, Tokyo, Japan) was used to characterize the AuNPs. A nano particle analyzer (Zetasizer Nano ZS90, Malvern Instruments Ltd., Malvern, UK) was utilized to measure the AuNPs size. An ultrasonic cleaner (KQ-500B) and ultrasonic cell crusher instrument (BILON92-ⅡDL) were purchased from Bilon Instruments Co. (Shanghai, China). A gas chromatography-mass spectrometry (Agilent 6890N-5973N, Santa Clara, CA, USA) was used to determine the CAP residues in fish samples.

### 2.3. Synthesis of Colloidal Gold

Colloidal gold nanoparticles (AuNPs) with a mean diameter of about 20 nm were prepared by chemical reduction of HAuCl_4_ with sodium citrate [28]. Briefly, an aqueous solution of chloroauric acid (50 mL of 0.01% (W/V) HAuCl_4_·3H_2_O) was heated to boiling point (110 °C), followed by the addition of 2 mL 1.0% (W/V) sodium citrate solution. The mixed solution was stirred simultaneously and kept boiling until the color of the solution changed from straw yellow to scarlet red. The surface of citrate-stabilized AuNPs has good performance for strong adsorption of antibody due to three types of interactions, including hydrophobic interactions, ionic interactions and dative binding. AuNPs-antibody conjugates are drawing more and more attraction in immunostrips and electrochemical immunosensors, due to their specific affinities to the target antigens.

### 2.4. Preparation of Colloidal Gold Probes

Polyclonal CAP antibody solution (1 mg/mL) was prepared by using sterilizing PBS (0.001 mol/L, pH 7.4), then was put into a dialysis bag for purification, and dialyzed at 4 °C in 0.005 mol/L NaCl aqueous solution. The dialysis filtrates were replaced 2–3 times at the initial stage of the dialysis process to remove the excess salt ions. After an overnight dialysis, the obtained solution was centrifuged at 10,000 rpm for 60 min at 4 °C to get a clear supernatant for conjugation. The optimal pH of the colloidal gold for antibody conjugation was adjusted by adding drops of 0.1 mol/L K_2_CO_3_ aqueous solution. With gentle stirring, antibody solution was added drop by drop to the colloidal gold solution (20 mL). After reaction for 30 min, PBS containing 10% BSA was added until the final concentration was 1%. The solution was allowed to react for another 15 min, and then was mixed with 5% PEG20000 until the concentration became 0.1%. In order to remove the excess antibody, the reaction mixture was centrifuged at 1500 rpm for 10 min and then at 13,000 rpm for another 60 min at 4 °C. The clear supernatant was carefully removed, and gold pellets were re-suspended in storage buffer (1% BSA, 0.01 mol/L PBS with 0.05% PEG20000, pH 8.0). The centrifugation was repeated 2 to 3 times, and then stored at 4 °C until use.

### 2.5. Optimization of pH for the Conjugation

pH is one of the key factors affecting colloidal gold conjugate. In this study, 500 μL colloidal gold solution was put into an Eppendorf tube with 0, 10, 20, 40, 60, 80 and 100 μL 0.1 mol/L K_2_CO_3_ added to obtain solutions of different pH values. While gently stirring, 1 mg/mL antibody solution (5 μL) was added dropwise to the obtained solutions (200 μL) respectively. After allowing the solutions to react for 10–15 min, each tube received 50 μL of 10% NaCl. Five min later, the absorbance of each solution was measured using the UV spectrometer.

### 2.6. Preparation of Immunochromatographic Strip

**Figure 1.**
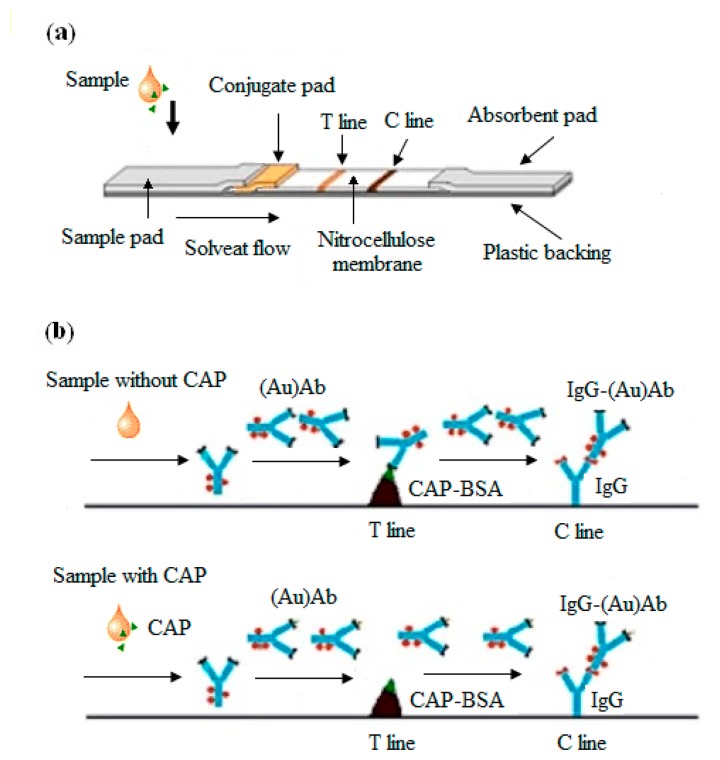
(**a**) The structure of the as-prepared strip; (**b**) Principle of gold immunochromatographic assay.

The preparation of the strip was conducted as follows: first, a nitrocellulose (NC) membrane was directly mounted onto the center of a PVC backing card. Second, a colloidal gold-labeled antibody conjugate pad was put onto one end of the NC membrane. An absorbent pad was then added to the other end of the NC membrane. Once that was done, a sample pad was put onto the side of the conjugate pad that was far away to the NC membrane. After that, the whole strip was flattened so that the sample liquid could flow successfully through the various materials on the strip. Finally, the CAP-BSA and goat anti-rabbit antibody were then separately sprayed onto a test line (T line) and a control line (C line) in the NC membrane. The structure of the obtained strip is shown in Figure 1a.

The operational principle of the developed test strip was described in Figure 1b as follows: the sample solution was pipetted onto the sample pad and gold-labeled antibody was added on the gold conjugate pad. The antibody would migrate upward due to the action of capillary forces. When colloidal AuNPs-antibody conjugates reached the test line, the coated antigens would compete with the analyte antigens in the sample for binding onto specific sites on the gold-labeled antibody. If there were fewer antigens in the sample, more gold-labeled antibody would be captured in the T line, so that the T line showed up as a red band (negative result). Otherwise, the colorimetric signal generated by the captured AuNPs would decrease as the concentration of analyte antigens increases, and thus the T line didn't become red (positive result). The competitive immunoassay generally has greater precision, accuracy, and reproducibility than noncompetitive (sandwich) assays [29].

## 3. Results and Discussion

### 3.1. The Quality Identification of Colloidal Gold

**Figure 2.**
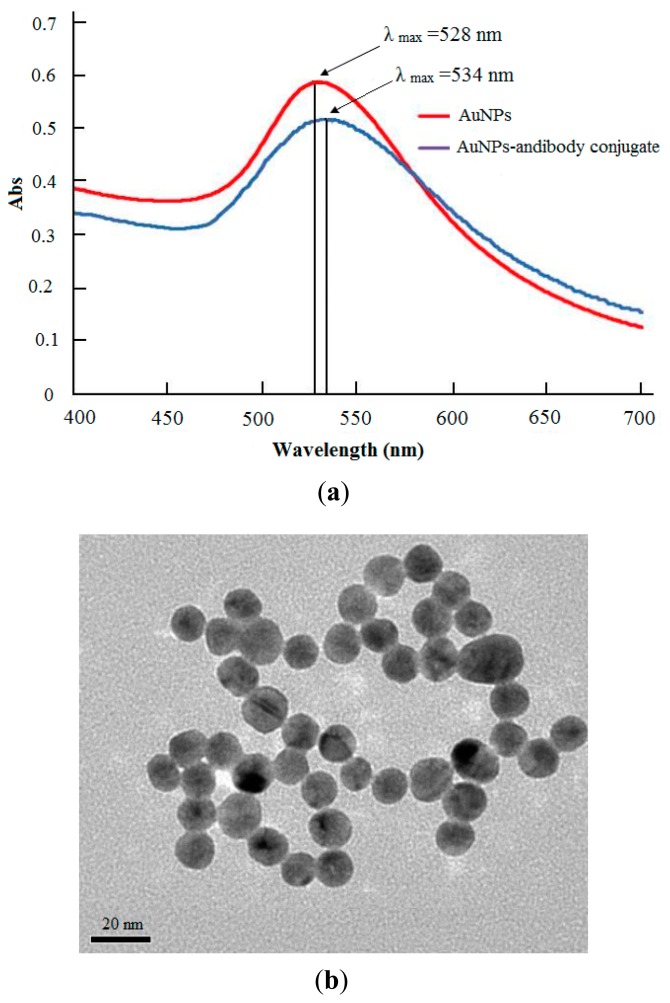
(**a**) The spectra of colloidal gold (red line) and antibody-colloidal gold conjugate (blue line); (**b**) TEM image of 20 nm gold nanoparticles.

The prepared colloidal gold solutions were claret red. A UV-spectrophotometer was used to characterize the obtained AuNPs based on their peak wavelength. The results showed that the wavelength of the maximum absorption peak was at 520 nm and the width of the main peak was small (Figure 2a, red line). It implied that the uniformity of colloidal AuNPs size was good and the size of AuNPs should be about 15–20 nm [30]. After the AuNPs were conjugated with the CAP antibody, the absorption peak shifted a little toward the red direction, and the main peak became wider while the maximum absorption peak became smaller (Figure 2a, blue line). This indicated that the diameter of antibody-colloidal gold conjugate became larger and the AuNPs size became slightly less uniform.

In addition, the TEM image of well-dispersed colloidal AuNPs, as shown in Figure 2b, established that colloidal gold particles were relatively uniform in size. The diameter of AuNPs was about 20 nm. This result was coincident with that from the UV-vis spectra. A quantitative approach was also used to measure the particle size distribution by using Zetasizer Nano ZS90. The statistical calculation result of one-hundred gold particles displayed that the average size of AuNPs was 20.7 nm with a polydispersity index of 0.20, implying the obtained AuNPs with uniform size.

### 3.2. Optimization of Colloidal AuNPs-Antibody Conjugates

#### 3.2.1. Optimal pH Value for the Conjugation

**Figure 3.**
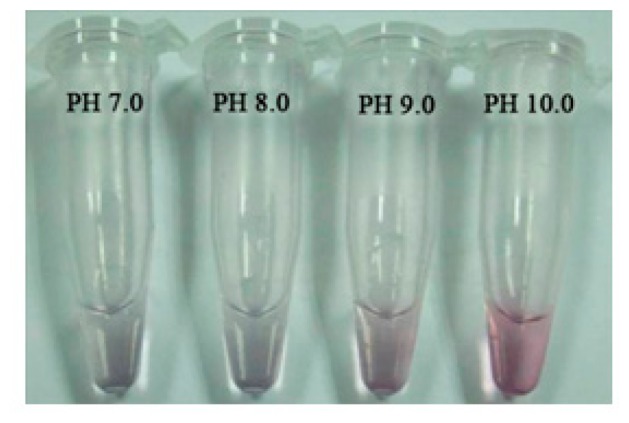
Different pH for the conjugation between colloidal gold and antibody.

The absorbance measurement results of AuNPs-antibody conjugates solutions at different pH values were listed in Table 1. Based on the data in Table 1, a curve was drawn by using the absorbance at 525 nm (OD_525_) as vertical coordinate and the K_2_CO_3_ concentration as horizontal coordinate (data not shown). The maximum OD_525_ was reached when 500 μL colloidal gold solution was added with 20 μL 0.1 mol/L K_2_CO_3_. The pH value of this mixed solution was considered to be the optimal pH, and was found at 8.0 using an accurate pH test paper. Figure 3 showed the obtained conjugates solution samples at different pH values.

#### 3.2.2. Optimal Marker Protein Amount for the Conjugation

In order to stabilize the colloidal gold particles, a minimum amount of antibody is required. The optimization procedure was conducted as follows. First, different amount of the antibody solution (in ascending order from 0 to 50 μL) was respectively immersed into 500 μL colloidal gold solution containing different amount of PBS (0.001 mol/L, pH 7.4, in descending order from 100 to 50 μL), and mixed for 15 min. Then, 100 μL NaCl (10%) was quickly added and blended. After that, the absorbance of the mixture was measured at 525 nm after 15 min. The results suggested that colloidal AuNPs-CAP antibody conjugates were synthesized successfully when absorbance became stable.

**Figure 4.**
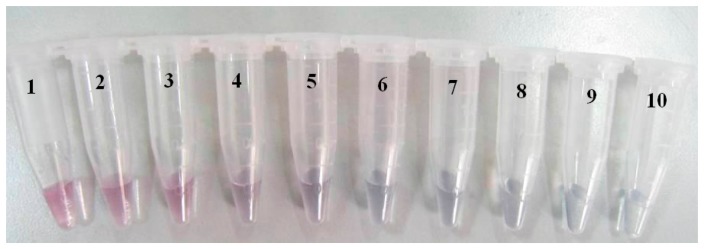
Optimal amount of marker protein for the conjugation.

Table 2 showed the absorbance of colloidal AuNPs labeled with different amount of antibodies. Then a curve was drawn through using the amount of antibody and OD_525_ as the horizontal and vertical coordinate respectively (data not shown). The amount of antibody corresponding to the point mostly close to *x* axis was selected as the minimum value for stabilization of colloidal gold. It was found that 3.0 μg of polyclonal antibody of CAP was confirmed to be the minimum amount, instead of 4.0 μg due to the cost control. The optimal amount for practical application can be set as 120% of the minimum value, that is 3.6 μg antibodies for 500 μL colloidal gold (*i.e.*, 7.2 μg antibodies for 1 mL colloidal gold). Figure 4 showed the obtained samples of colloidal AuNPs labeled with different amount of antibody. The sample in the tube with No.6 was the optimal AuNPs-antibody conjugate.

### 3.3. Optimization of the Strip Test

#### 3.3.1. Pretreatment Optimization of the Conjugate Pad

It is important to choose a suitable membrane for the conjugate pad, and fiberglass membrane seems to be a good choice because of its hydrophilicity and complete dilution. In this study, a fiberglass membrane was cut into small 5 mm × 8 mm pieces. These pieces were then put into 0.01 mol/L phosphate-buffered saline (PBS, pH 8) with 0.05% Tween-20 under the conditions of 5%, 10%, 20% sucrose solution. Finally, it was taken out after 10 min, and then dried at room temperature for later use. Through observing the lateral flow speed and color intensity change of gold-labeled antibody during the test procedure (data not shown), it was found that the most ideal results were obtained when the content of the sucrose was 10%. Consequently, PBS (0.01 mol/L, pH 8.0) containing 10% sucrose and 0.05% Tween-20 was considered to be the optimal pretreatment solution for the conjugate pad.

#### 3.3.2. Optimization of Blocking NC Membrane

It is well known that any excess antibody present will compete for binding sites on the surface of labeled gold colloid and hence lower the sensitivity of the detector reagent in the assay. Here, BSA was used to block the remaining active sites on the membrane. First, 1 mg/mL antigen (T line,) and 10 mg/mL goat anti-rabbit antibody was separately spotted on T line and C line on the NC membrane, and placed at room temperature for 2 h. After that, the above NC membrane was immersed into PBS (0.001 mol/L, pH 7.4) containing BSA of different concentration (0.5%, 1%, 3%), blocking at 37 °C for 30 min. Finally, the NC membrane was dried at 37 °C for later use.

**Figure 5.**
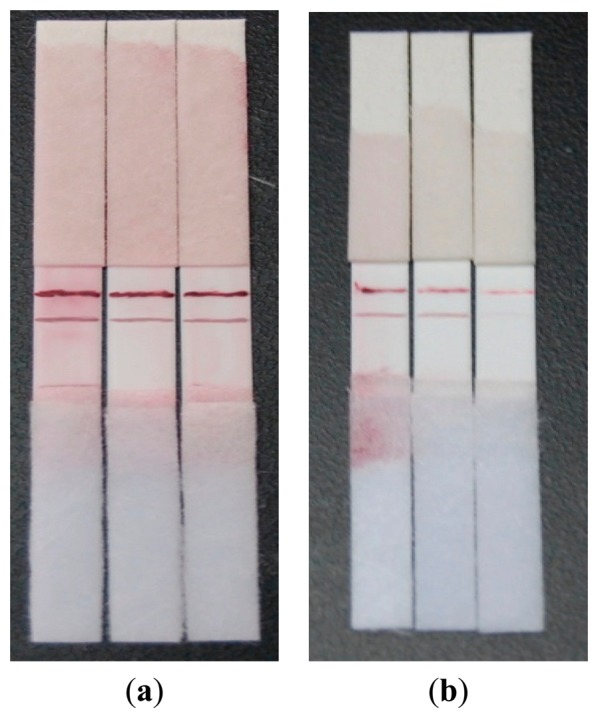
(**a**) Test results of the strips blocked by BSA with different concentration of 0.5%, 1% and 3%, from left to right; (**b**) Test results of the strips immobilized with antibody–colloidal gold conjugates diluted 2, 5, 10 times, from left to right.

As shown in Figure 5a, it can be seen that the background color of the test strip was almost white and easy to differentiate when the concentration of BSA in blocking buffers was 1%, which indicated that the protein in blocking buffers was sufficient for blocking non-specific binding sites on the NC membrane. However, higher concentrations of BSA containing more protein weakened the color intensity of lines. Hence, PBS with 1% BSA was recommended as a suitable blocking material for strips.

#### 3.3.3. Optimization of the Amount of Gold-Labeled Antibody

The gold-labeled antibody solution (see Section 2.4) was diluted 2, 5 and 10 times with buffers (0.01 mol/L PBS; pH 8.0) containing 1% BSA and 0.05% PEG20000. 20 μL of different diluted antibody solutions was respectively transferred onto a pretreated nitrocellulose membrane; which was then used in the strips. As shown in Figure 5b, using PBS as a test sample, when the gold-labeled antibody solution was diluted twice, the amount of gold-labeled antibody that was conjugated was more than that could be absorbed by capillary action, which resulted in the residue of gold-labeled antibody on the conjugate pad and reinforced the background color. When diluted 10 times, it was not enough to finish the line coloration. Therefore, the gold-labeled antibody solution diluted five times was the optimal choice, due to the ideal effect of lines coloration.

#### 3.3.4. Optimization of the Concentration of the T Line and C Line

**Figure 6.**
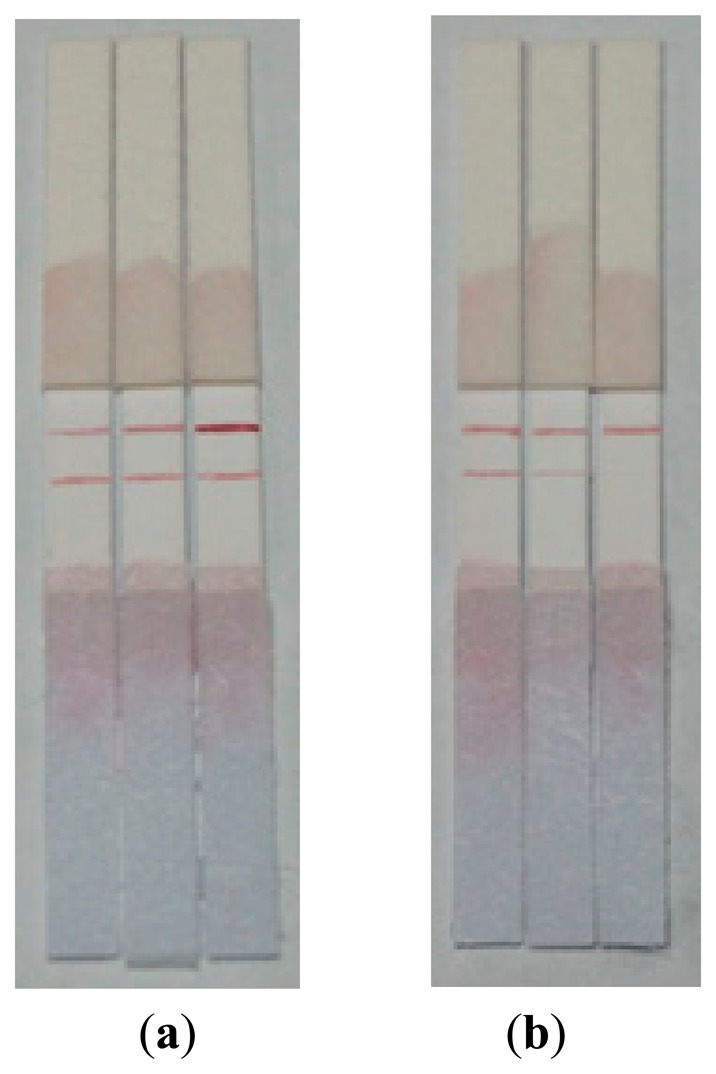
(**a**) Results on 1, 0.5 and 0.2 mg/mL of IgG (from left to right) under 1 mg/mL concentration of immobilized antigen conjugate; (**b**) Results on 1, 0.5 and 0.2 mg/mL of immobilized antigen conjugate (from left to right) under 0.5 mg/mL concentration of immobilized IgG.

The C line was coated with 1 μL goat anti-rabbit antibody with a concentration of 1 mg/mL, 0.5 mg/mL, 0.2 mg/mL diluted by PBS (0.01 mol/L, pH 7.4) respectively, and the T line was coated with 1 mg/mL antigen conjugate. Then, through observing the color of strips by testing PBS, the optimal concentration of goat anti-rabbit antibody could be determined. After that, the concentration of antibody (T line) could be decided in the same way. As shown in Figure 6a,b, 1 mg/mL, 0.5 mg/mL was regarded as the best concentration of T line and C line respectively, because the line color was easy to distinguish with the naked eye.

### 3.4. Characterizations of Strips

#### 3.4.1. Sensitivity of Strips

Standard solutions containing 0, 0.2, 0.5, 1 and 2 ng/mL CAP respectively were used to detect the sensitivity of strips.

**Figure 7.**
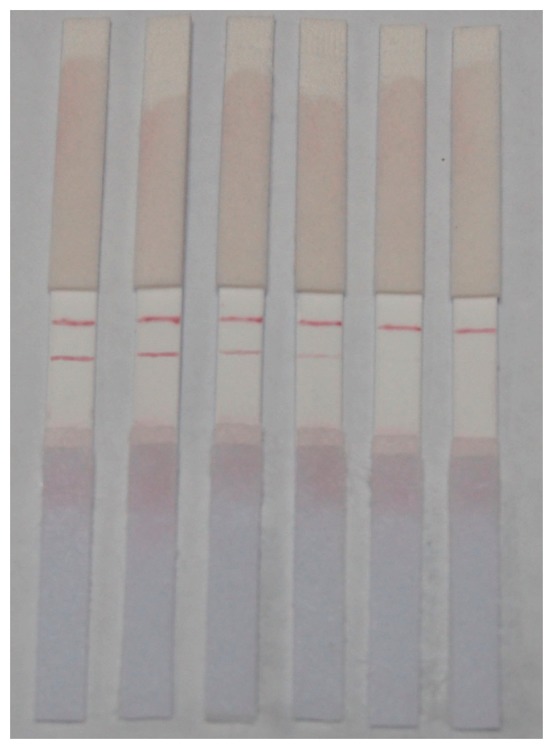
Results on the sensitivity of strips (the concentrations of CAP in the solutions is 0, 0.2, 0.5, 1 and 2 ng/mL respectively, from left to right).

Different concentrations of standard solutions were dropped onto the prepared strips. When the solutions reached the NC membranes (after about 30 s), the strips were flattened, and 10 min later images were produced. Results are shown in Figure 7. The minimum detectable concentration of CAP using these strips was defined as the concentration of CAP in the solution that caused the color of T line to become obviously more invisible than that of the C line, and that was 0.5 ng/mL with good reproducibility, which was confirmed by ten tests.

#### 3.4.2. The Analysis Time of Strips

Ten of the prepared strips were chosen at random, and were used to test the time of lateral flow process (the time it took for sample solutions pipetted onto the sample pad to migrate upward to NC membrane) and lines coloration time (the time for the produced change in color intensity of the test line (signal intensity)). After ten measurements, results showed that the time of lateral flow was about 30 s, and the line coloration time was 9.3 (±1.2) min. Hence, the analysis time for assay of CAP was within 10 min. Compared to the methods of gas chromatography [10,11], liquid chromatography [13–15] and enzyme-linked immunoassay [16], the strips had the benefits of faster results (the entire process not exceeding 10 min), relative ease of use and no technical expertise required to perform the tests. Thus the method can be used for fast on-site detection of CAP residues in aquatic products.

#### 3.4.3. Reproducibility of Strips

In order to evaluate the reproducibility of strips, 20 strips were randomly selected from different batches, and were used to detect negative and positive sample solutions. Each solution was measured ten times.

The positive samples were from 1.5 ng/mL of CAP standard solution, and the negative samples were from 0.3 ng/mL of CAP standard solution. Three groups (ten samples in each) were collected to evaluate the reproducibility of the tests. Experimental results showed that strips could easily discriminate between negative and positive samples, and did not falsely display the T line for any of the negative samples.

#### 3.4.4. The Stability of Strips

A batch of strips were prepared and divided into three parts, then sealed and separately stored at 4 °C, room temperature and 37 °C. Two strips of each part were assessed every 30 days by testing the negative and positive solution samples respectively in order to evaluate the stability of strips. Results demonstrated that the performance of strips at different temperatures was nearly the same, and strips had a stable storage life of more than 90 days.

#### 3.4.5. Detection of CAP Residues in Real Samples

**Figure 8.**
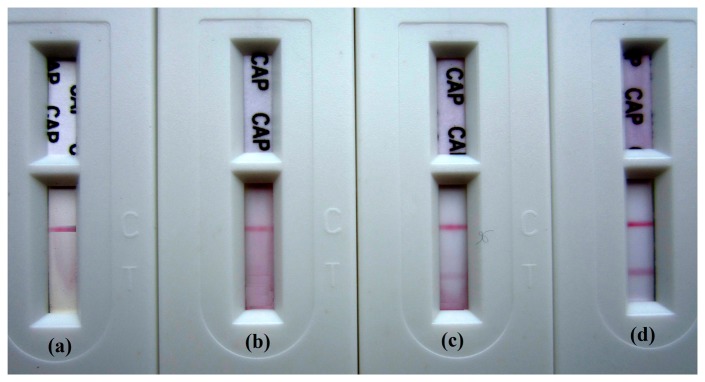
The assay results of crucian carp muscles injected with CAP at dose of 5, 3, 1 and 0 μg/kg.bw respectively, from left to right.

Samples of crucian carp were tested using the prepared colloidal gold immunochromatographic strips. For convenience in practical application, the strips were installed with shells (see Figure 8). A stock solution of 1 mg/mL CAP was prepared in methanol and water (1:9, v/v) and stored at 4 °C. It was diluted to the desired concentrations before use. Eight healthy crucian carp (*Carassius auratus*) weighing between 180 and 200 g were purchased from a local market. Fish were cultivated in eight water tanks under standard conditions. Two of them were named the control group, and the remaining pairs were injected with CAP solution in their dorsal muscles at doses of 1, 3, 5 μg/kg.bw respectively. After 9 h from the time of injection, the fishes were collected, killed, and their dorsal muscles were dissected, weighed and stored at room temperature. After that, eight samples of 2 g muscles from individual fish was separately homogenized using the ultrasonic cell crusher with 3 mL methanol and 1 mL PBS (pH 7.4) for 10 min, and then quickly filtered through filter papers. The filtrates were ready for assay using the strips. Above all, the preparation of samples from individual fish can be finished within 20 min.

It was found that the T line disappeared on the strips for detection of fishes injected with CAP at a dose of 5 μg/kg.bw (see Figure 8a), since the concentration of CAP residue far exceeded the negative concentrations detected by the strips. Fishes injected with CAP at a dose of 3 and 1 μg/kg.bw caused a relatively distinguishable difference (see Figure 8b,c), when compared to the negative control. For fishes injected with no CAP, the color of T lines on the strips was nearly unchanged, and there was no distinction compared to the color of C lines (Figure 8d). As reported, the elimination half-life (*t*_1/2_) of CAP for carp muscles (*Cyprinus carpio L.*) was 9.28 h [31]. According to that report, we can know that after 9 h from the time of injection with CAP at a dose of 1 μg/kg.bw, the concentration of CAP residue in carp muscles was about 0.5 μg/kg.

**Figure 9.**
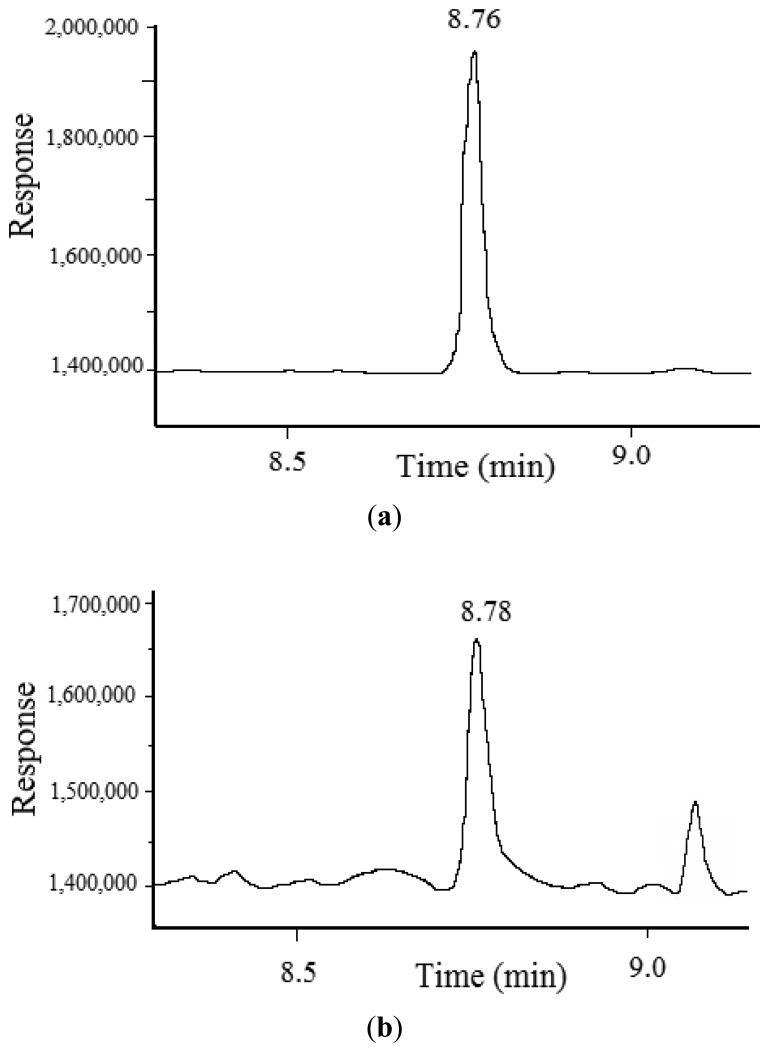
The chromatogram of CAP: (**a**) standard, 1 μg/kg; (**b**) carp muscles from the fish injected with CAP at a dose of 1 μg/kg.bw after 9 h from the time of injection.

In order to analyze the actual concentration of CAP residue in fish muscles, the standard GC-MS method [32] was used to validate the measured results by test strips. The GC-MS instrument was equipped with a micro-electron capture detector (μ-ECD) and a capillary column (Agilent DB-35MS, 30 m × 0.25 mm × 0.25 μm, Santa Clara, CA, USA). Helium was used as the carrier gas at a flow rate of 1.5 mL/min and nitrogen was used as make-up gas at a flow rate of 50 mL/min. The temperature profile was programmed to hold an initial temperature of 150 °C for one min, followed by a gradient of 20 °C/min up to 260 °C, where it was held for 10 min. The temperatures of the injector and detector were set at 260 °C and 280 °C respectively. The volume of sample was 1 μL. The samples were prepared with ethyl acetate as extractant, the mixture of n-hexane and NaCl as degreasing agent, and N,O-bis(trimethylsilyl)trifluoroacetamide as derivatization agent. The results verified that the CAP residue in carp muscles was about 0.48 μg/kg (see Figure 9b). Therefore, it is recommended that the minimum detectable concentration of CAP residue in fish using test strips is 0.5 μg/kg, which is effective for practical application. Simultaneously, the practical test can be finished within half an hour, including samples pretreatment (about 20 min) and chromatographic analysis (less than 10 min).

In addition, the sensitivity of test strips was compared with that of the standard reference methods. A gas chromatography method with microcell electron capture detection was developed for the determination of CAP residue in fish and shrimp muscle tissues [11]. The limit of quantification was 0.1 μg/kg. Analytical methods based on gas chromatography-mass spectrometry (GC-MS) and liquid chromatography-mass spectrometry (LC-MS) were developed for the determination of chloramphenicol (CAP) levels in milk. The detection limit for CAP by LC-MS and GC-MS was 0.15 μg/kg and 0.14 μg/kg respectively [33]. A high-performance liquid chromatography coupled with quadruple mass spectrometry was validated for CAP quantification at the concentration range from 0.30 to 3.00 ng/mL [13]. Apart from that, the enzyme-linked immunoassay (ELISA) could detect chloramphenicol at levels below the minimum required performance limit of 0.3 μg/kg [34], but the assay duration was in the range of 1–2 h. Compared with these reported methods, the strips presented in this study can detect the minimum concentration of CAP residue in fish muscles as low as 0.5 μg/kg within half an hour including samples pretreatment. However, this is only a qualitative method. The concentration value of CAP residues in fish can't be given by the proposed strips. Nevertheless, the concentration of CAP residue in fish that is higher than 0.5 μg/kg can be judged by this strip, so it provides a useful tool for on-site fast screening of CAP residues in aquatic products.

## 4. Conclusions

A colloidal gold immunochromatographic strip was developed for rapid detection of chloramphenicol residues in aquatic products. A NC membrane was used as the carrier, and polyclonal CAP antibody was used as the marker protein, and goat anti-rabbit antibody was used as the conjugate secondary antibody. The minimum detectable concentration of CAP residue in fish muscles using this strip was as low as 0.5 μg/kg, with good reproducibility. The as-prepared strip is more sensitive than the previously reported test strips based on colloidal gold. Moreover, the chromatographic analysis time was less than 10 min, and the strips had a long shelf life of more than 90 days for use in practical applications. The strips provide a potential pathway for rapid, sensitive on-site detection of CAP in aquatic products.

## Figures and Tables

**Table 1.** Optimization of pH for the conjugation between colloidal gold and antibody.

**Tube Number (No.)**	**The Amount of 0.1 mol/L K_2_CO_3_ (μL)**	**OD_525_**
1	0	0.2185
2	10	0.1559
3	20	0.2275
4	40	0.1844
5	60	0.2119
6	80	0.1774
7	100	0.1853

**Table 2.** The absorbance of colloidal AuNPs labeled with different amount of antibody.

**Tube Number (No.)**	**Polyclonal Antibodies of CAP (μg)**	**OD_525_**
0	0	0.3615
1	0.5	0.3332
2	1.0	0.3122
3	1.5	0.3083
4	2.0	0.2740
5	2.5	0.2576
6	3.0	0.2351
7	3.5	0.2412
8	4.0	0.2303
9	4.5	0.2396
10	5.0	0.2379
